# The Institutional Worker

**Published:** 1913-05-24

**Authors:** 


THE
### INSTITUTIONAL WORKER
**Being a Special Supplement to "The Hospital"**

### OUR BUREAU OF INFORMATION.

### Rules for Correspondents.

1. Every letter must be accompanied by the coupon to be cut from the back cover (inside page) of The Hospital, current issue, and must contain the name and address of the correspondent with pseudonym for publication if desired. Replies by post cannot be given, save under exceptional circumstances at the Editor's discretion.

2. Letters from Approved Homes in reply to special needs published in the Bureau should state terms and full particulars, and be sent prepaid under cover to the Editor of the Bureau with name written across coupon for identification.

3. Proprietors of Homes which have not yet been entered on the List of Approved Homes, but have spare accommodation likely to suit special needs, are invited to write for an application form for registration. The fee for registration, which includes two announcements of the Home in the Bureau and other privileges, is 10s.

4. All communications to be addressed to the Editor of The Hospital, 28 Southampton Street, Strand, London, W.C., and marked "Bureau of Information."

### *THE SICK AND DISTRESSED.*

**The Editor is prepared to make known without charge the needs of any who may be sick or in difficulties, and to guide them in making choice of Homes for treatment or convalescence. Reduced terms can often be arranged with Homes on the Approved List for those unable to pay full fees. Queries are specially invited from those who are engaged in any kind of philanthropical work. A new edition of the leaflet describing the purposes of the Bureau of Information is now ready and will be sent free of charge on application.**

### Personal Service and Co-operation.

The power of personal service in the relief of distress and suffering has been so often emphasised in these columns that we feel sure that our readers will be gratified and encouraged to hear of an instance where co-operation between them and our Bureau has been the means of lightening the load of affliction of one poor institutional worker.

Blind and Deaf.

Many of our readers have taken an interest in this case. An attack of typhoid fever, contracted in the wards of one of the Metropolitan Asylums Board institutions, left this energetic worker stone deaf and so nearly blind that she is unable to venture out alone and retains a mere glimmer of light. On her tiny pension she lives with an aged mother, whose sole source of income is the old age pension. Together they supplement each other's deficiences of strength, sight, and hearing, but the struggle has been a severe one. It was realised as soon as the circumstances were brought to our notice that an increased income was the only way of alleviating the lot of these brave women, who had managed to keep an exquisitely neat home round them by dint, it is to be feared, of undergoing many privations in warmth and food.

The National Blind Relief Society.

An effort to procure a pension from one of the numerous societies for the relief of the blind was attended by constant disappointments, and it became evident that there were many more blind persons in need of succour than existing funds for their relief. At length, however, our attention was drawn to "The National Blind Relief Society," where small pensions are promised within a year of the receipt of subscriptions amounting to at least two guineas for special cases. Within a short time two subscribers were found to guarantee a guinea each for the relief of our friend, and a sum was raised to provide a small monthly allowance until the pension should become due. The National Blind Relief Society has, however, been far better than its word. Two gifts of one pound have been already forwarded from the Secretary, the Rev. J. Pullein-Thompson, and the news now reaches us that a pension amounting to 10s. a month has been granted, and this within six months of the application. Very cordial thanks are due to Mr. Thompson for the interest he has taken in this afflicted hospital worker. He has made independent efforts on her behalf which have interested other friends in her, and by this means, instead of waiting her turn, she enters at once upon the allowance she sorely needs. She has been able, through the assistance already granted, to move to more suitable quarters, and her general health has greatly improved owing to the cessation of wearing anxiety about ways and means.

### Case of Advanced Phthisis.

If the poor woman suffering from advanced phthisis is a member of the Church of England, you might apply to the Sister-in-Charge, St. Michael's Home, Axbridge; otherwise we are unable at present to suggest a Home suitable for the case. We are making further inquiries, and meanwhile have announced your requirement in these columns.—"H. E. B."

### Consumptive Home Required.

Could any of our readers help us to find a Home for a young woman suffering from phthisis in an advanced stage, who has been deserted by her husband? Several efforts have been made to get her into a sanatorium, but her condition is regarded as too hopeless. She is at present living with her parents and three other members of the family in a small cottage near Shrewsbury. As they are very poor nothing can be paid towards the sufferer's maintenance. Prior to her illness she was employed as a housemaid. Such assistance as any of our readers can render will be gratefully welcomed. Replies should be addressed H. E. B., 160b.

### Home of Rest Required.

M. S. writes: Can anyone tell me if there is a Home near London where a lady's maid could go whose condition of health necessitates a long rest; she is unable to contribute anything toward her maintenance? Possible some of our readers could help us in this matter, and we should be glad to hear from any of them; or from any charitable agency able to assist. Meanwhile we would suggest that as the case is one which might be eligible for treatment at a convalescent home, application should be made to the Metropolitan Convalescent Institution, 14 Victoria Street, Westminster. Admission to the homes of the Institution is free upon a subscriber's recommendation.

### Approved Homes.

CHINGFORD.—42 The Avenue, Highams Park. Principal: Miss Hilda Wood (Medico-Psychological certificate). A comfortable Home for those suffering from mild mental and nervous diseases, and for chronic invalids. Terms, from 21s. a week.

There is a vacancy at one of our Approved Homes for a permanent case at £3 3s. a week. Apply, C. S. T., 161a.

### *THE INSTITUTIONAL OFFICER.*

### Who Should Provide the Ambulance?

It frequently occurs that the general hospitals are appealed to by the friends or relatives of sick and injured persons to send an ambulance for the removal of some poor sufferer to the hospital. Very seldom, we fear, is such an appeal responded to in a practical manner. Comparatively few hospitals in this country possess an ambulance, and the most that hospital officers can do is to suggest that the patient be brought in a cab or some similar conveyance.

Needless Suffering.

Were we to watch the admission departments of some of our provincial hospitals we would see the most desperate cases being brought up for treatment in all sorts of conveyances at the cost of great suffering and grave risk to life. The contemplation of these facts raises the question asked in the headline of this note. The local authorities, in conjunction with the police, deal with the cases of accident or sickness occurring in the streets. But how many of the smaller towns possess an ambulance service? The police certainly do their best with the hand ambulances at their disposal, but when the seriousness of the case demands its speedy treatment they are obliged to make use of a cab. Even in the Metropolis the service is not perfect, as has been pointed out by the City Coroner. In the industrial areas the proprietors of large works and collieries have shown a greater appreciation of the need than either the hospital or local authorities by the provision of private ambulances.

A SUGGESTION.

The arrangement which has recently been entered into whereby the London County Council has agreed to contribute £300 a year toward the maintenance of a motor ambulance presented by the Grand Duke Michael to the Hampstead General and North-West London Hospital seems to suggest that the matter is one upon which the hospital and local authority might join hands in dealing with. Doubtless there are many hospital officers to whom this great need has presented itself, who have considered the matter and have some comments and suggestions to make which will be of interest to our readers.

### *INSTITUTIONAL FACTS AND FIGURES.*

### QUESTION FOR MAY.

**During a period of four weeks in the winter months state what was the quantity of gas consumed in your institution for purposes other than illuminating, its approximate cost, the quantity of each kind of fuel used and its cost, deducting the cost of fuel for laundry purposes (if any).**

**In answering this question the following particulars should be given: (1) Number of wards and size of each (in beds); (2) total number of coal fires (a) in the wards, (b) in the rest of the building; (3) the number of gas-cooking and other stoves and sterilisers; (4) the name of the heating and hot-water supply service.**

**In the ensuing month contributors will be asked to give fuller particulars upon this subject by describing the character of the buildings, apparatus, etc.**

NOTE.—The figures must be the result of actual observation and computation, not estimates. The best answers will be published, and for each contribution considered by the Editor to be of sufficient interest for publication payment of FIVE SHILLINGS will be made.

RULES.

The following rules must be observed by all those answering the Facts and Figures Questions:—

1. Answers must be written on one side of the paper. They must bear the name and address of the sender and be accompanied by coupon to be cut from the back cover (inside page) of the current issue of THE HOSPITAL. A pseudonym must be chosen if the name is not to be published.

2. Answers must be addressed to the Editor of THE HOSPITAL, 28 & 29 Southampton Street, Strand, London, W.C.; they must be marked in the left-hand corner "Facts and Figures," and must be received before the end of the month.

### Vapour Bath.

A VAPOUR bath in bed is managed in the same way as a hot-air bath, the only difference being that steam is introduced into the bed instead of hot air. If you have not the special apparatus, you can make use of a kettle with a long spout, such as a bronchitis kettle. See that the mackintosh is well arranged over the cradle, or the bed clothes will get very damp. Twenty minutes is usually allowed for a vapour bath. Hot bricks wrapped in a wet blanket, and placed round the patient beneath the cradle, will answer the same purpose.—"NOVICE."

### Economy in Hospital Dressings.

THE use of washed bandages is very often objected to by surgeons, and the reasons for the objection are, no doubt, that the washing is badly done, and the bandages are not properly straightened out and rolled before being sent to the respective departments for use. Great economy can, however, be effected by washing and sterilising gauze and bandages that have already been used provided the hospital possesses the necessary conveniences for carrying out the work effectively. The following method is described by the superintendent of one of the American hospitals, and we believe that a similar process is practised at the Glasgow Western Infirmary. Gauze and bandages from ward dressings, and operating rooms are sent to a sorting room in bags. Bandages are separated from gauze, packed loosely in net bags, which are placed in tubs, covered with cold water, and left to soak overnight. In the morning they are placed in a washer capable of resisting 10 lb. pressure of steam; they are washed first in cold water for ten minutes; drained, and washed in cold water with soap solution and soda for fifteen minutes. They are then drained again; warm water is turned into the washer, to which is added soap solution and soda. The washer is then sealed and 10 lb. of live steam is turned on for thirty minutes, followed by a hot rinse for fifteen minutes and a cold rinse for fifteen minutes. The gauze, etc., is then put in the hydro-extractor, and, when dry, carefully overhauled, untangled, and straightened, and placed in packages for the last sterilisation, which is conducted by the theatre nurse. Washed gauze is softer and more absorbent than new gauze.—"MATRON."

### *THE INSTITUTIONAL HOUSEKEEPER.*

### Poultry Farming.

THE advantage to institutions of making use of waste ground for the purpose of keeping poultry, to which reference was made in the columns of THE HOSPITAL on May 10, is instanced by the following account which we have received from the Matron of the Bury Infirmary. Miss Farrow writes: The committee here allowed me to start a small pen of hens three years ago. It has reduced our egg bill considerably. At present we have twenty-one pullets and two male birds. The balance in January 1913 showed: Profit, £5 6s. 4½d.; value of stock, £3 10s.; total, £8 16s. 4½d. We had 238 eggs during April and 81 in December 1912. Though this is poultry farming in a very small way, the accounts show it is quite profitable, and we have the advantage of fresh eggs.

### *EMPLOYMENT AND TRAINING.*

### Scarcity of Private Nurses.

WE regret to hear that you have so far been unsuccessful in obtaining the trained nurses you require. You do not say whether you have applied to any of the Poor-Law infirmaries. As regards your need for trained masseuses, the Incorporated Society of Trained Masseuses, 12 Buckingham Street, Strand, W.C., acts as a registry for its members, and you might with advantage apply to the Secretary. Failing these sources, we can only suggest that you continue to advertise.—"E. A. S."

### Holiday Engagement.

YOU do not state in what capacity you desire a holiday engagement at a hospital, whether as chaplain or secretary. Kindly let us have this information, and also say whether you require an honorarium for your services.—"B. R."

### Nursing in South Africa.

WE hope to be able to give you some information regarding nursing associations in South Africa and Rhodesia in a later issue. As, however, within the Union of South Africa the registration, etc., of trained nurses is controlled by the Medical Councils of the four Provinces of the Union, we suggest, meanwhile, your communicating with the Secretaries of these bodies at Capetown, Pretoria, Pietermaritzburg, and Bloemfontein respectively.—"BRADFORD."

### Mental Nursing.

WE cannot give postal replies; a coupon should be enclosed with each inquiry. If you are in need of an engagement as a mental nurse we would suggest that you study the advertisement columns of the nursing journals, such as *The Nursing Mirror.* A List of Private Homes is published by The Scientific Press, Ltd., 28 and 29 Southampton Street, Strand, London, W.C., but it is impossible for us to suggest Homes where mental nurses are likely to be required.—"E. R."

### Massage and Electrical Treatment.

PLEASE read the rules. A coupon should be enclosed with each inquiry. You cannot practise massage and electrical treatment without a proper training, and then you should only do so under the direction of a medical practitioner. You can obtain an excellent training at the National Hospital for Paralysed and Epileptic, Queen Square, Bloomsbury, London. Application should be made to the Matron; the fee for the course, including electricity, is £13 13s. There are other training schools, full information concerning which you can obtain upon application to the Incorporated Society of Trained Masseuses, 12 Buckingham Street, Strand, London, W.C. This Society conducts an examination twice yearly.—"MINNIE."

### *EDITOR'S LETTER-BOX.*

COMMUNICATIONS have been received and attended to from M. Asquith and Sister Hewitt. The Editor is indebted to Miss Farrow for her note on "Institutional Poultry Farming."

THE Editor will be glad to receive correspondence and to consider contributions upon all subjects relating to institutional work, and affecting the welfare of institutional officers.

### Editor's Notices.

**Contributions:**

Contributions should be written, or preferably typed, on one side of the paper only, and all articles sent in are accepted upon the distinct understanding that they are forwarded to THE HOSPITAL only.

The Editor cannot undertake to return MSS. not used, but when a stamped directed envelope is enclosed the MSS. may be returned if a special request is made.

Accepted articles and paragraphs of news will be paid for after publication at the scale rate.

**Address:**

To prevent delay all contributions and letters on editorial business must be addressed exclusively to the Editor, "The Hospital," 29 Southampton Street, Strand, London, W.C. It is important that this regulation shall be strictly observed.

**Correspondence:**

Correspondence on all subjects is invited. The name and address of correspondents must be given as a guarantee of good faith, but not necessarily for publication.

**Special Articles:**

Special articles are invited, and questions, inquiries, and paragraphs upon all matters relating to the work, administration, and management of general, special, mental, fever, cottage and convalescent hospitals, sanatoria, homes, institutions, societies and organisations for the treatment or care of the sick, injured and dependants of all classes. Every matter affecting the interests and welfare of the staff of all grades working in these institutions will receive special consideration.

**Administrative Medicine, Research and Items of News:**

Special terms will be paid for approved articles on subjects in this wide field by experts who have made some section of it their special study and interest. We wish to give prominence to every movement tending to advance accuracy of diagnosis, efficiency in treatment, and the development of modern methods to eradicate disease and promote the welfare of the suffering.

**A Bureau of Information:**

We invite inquiries and applications for information and help in providing for that numerous class of sufferers who require special care or house-room which they cannot obtain in their own homes or secure for themselves. **We cannot, however, prescribe, or recommend practitioners.**

**Books for Review:**

Publishers are particularly requested to send advance proofs of any new books of importance whenever possible, as the Editor has made arrangements to publish immediate reviews on a new plan.

**Photographs, Plans, Blocks, and Illustrations:**

It is requested that wherever possible MSS. may be accompanied by illustrations in any of the above forms. The name of the sender and of the article to which it belongs should be written on the back of each photograph, drawing, or block for purposes of identification.

**Local Papers:**

Newspapers containing reports or news paragraphs should be marked and addressed to the Sub-Editor.

**The Coupon System:**

A coupon will be found at the bottom of the third inside page of the cover each week. This coupon must be attached to every question or inquiry to which an answer is desired in THE HOSPITAL.

### The Workers' Help.

OUR purpose is to make this Supplement of THE HOSPITAL of the greatest possible service to every Institutional Worker. The term is employed in its widest sense, and includes all those individuals engaged in or associated with Charitable, Poor-Law, and Municipal Administration, as well as the Medical, Health, and Sanitary Services.

Although the section is primarily intended to help those seeking appointments, and numerous announcements of vacancies appear in its columns every week, it is felt there are also many other ways in which it can be of value. A number of pages have recently been added containing information which it is hoped will prove of assistance to many of our readers in their work, and in this you can help us and yourself by showing a lively interest in this section and offering such suggestions for its improvement as may occur to you.

### Manager's Notices.

**Letters relating to the publication, sale, and advertising departments of "The Hospital" must be addressed to The Manager, "The Hospital," The Hospital Building, 28 and 29 Southampton Street, London, W.C.**

**Advertisements:**

To ensure insertion, all advertisements for THE HOSPITAL must reach the Manager not later than Wednesday morning in each week. For scale of charges see pages v and 4.

**Special Rates** will be quoted for those institutions that agree to send all their vacancy, contract, and public notices to THE HOSPITAL each year, so as to make it a file of such announcements for ready reference.

**Subscriptions** may begin at any time, and are payable in advance. Cheques and Post-Office Orders should be crossed London County and Westminster Bank, Covent Garden Branch, and made payable to the Manager of THE HOSPITAL.

RATES OF SUBSCRIPTION (including Postage).

| *United Kingdom.* | | *Foreign and Colonial.* | |
|---|---|---|---|
| Three Months ... ... | 2s. 0d. | Three Months ... ... | 2s. 6d. |
| Six Months ... ... | 4s. 0d | Six Months ... ... | 5s. 0d. |
| Twelve Months ... ... | 6s. 6d. | Twelve Months ... ... | 8s. 6d |

*SUBSCRIPTION FORM.*

*Please send me* THE HOSPITAL *for..............months, commencing with your issue of.............................., for which please find enclosed............................*

*Name*..........................................................

*Address* ..........................................................

..........................................................

*Date*....................................

*To* .................................... *Newsagent.*

**Remittances:**

**It is especially requested that remittances be made by Cheque or Postal Order, and in halfpenny stamps only when the amount is under sixpence.**

